# Treatment-free survival after discontinuation of immune checkpoint inhibitors in mNSCLC: a systematic review and meta-analysis

**DOI:** 10.3389/fimmu.2023.1202822

**Published:** 2023-07-13

**Authors:** Yue Hu, Shan Liu, Lixing Wang, Yu Liu, Duohan Zhang, Yinlong Zhao

**Affiliations:** Department of Nuclear Medicine, The Second Hospital of Jilin University, Changchun, China

**Keywords:** immunotherapy, immune checkpoint inhibitor (ICI), meta-analysis, non-small cell lung cancer (NSCLC), objective response rate (ORR), treatment-free survival (TFS)

## Abstract

**Background:**

Recent research has suggested that patients with metastatic non-small cell lung cancer (mNSCLC) can achieve ongoing response after discontinuation of immune checkpoint inhibitor (ICI), but the best time to discontinue and the factors influencing efficacy remain unknown.

**Method:**

A systematic search was performed for prospective clinical trials in patients with mNSCLC treated with ICIs published up to July 10, 2022. Eligible studies reported treatment-free survival (TFS) after discontinuation of ICI in partial objective responders. We calculated objective response rate (ORR) and TFS using random-effects models with respective 95% confidence intervals (Cis), and performed subgroup analyses to discuss the specific associations between ORR and TFS and the associated influencing factors.

**Results:**

Across the 26 cohorts (3833 patients) included, the weighted mean ORR for all patients was 29.30% (95% CI 24.28% to 34.57%), with ICI plus chemotherapy (48.83%, 95% CI 44.36% to 53.30%) significantly higher than monotherapy (23.40%, 95% CI 18.53% to 28.62%). 395 patients were all patients who were complete or partial responders in the study, 194 discontinued ICI treatment, and nearly 35.5% achieved a durable response. No significant differences in TFS were found between subgroups according to the ICI regimen classification. Four cohorts of patients who completed 35 courses of treatment showed high levels of pooled TFS at 6 (80.18%, 95% CI 53.03% to 97.87%) and 12 months (66.98%, 95% CI 46.90% to 84.47%). Three cohorts of patients discontinued ICI treatment due to treatment-related adverse events (TRAEs) with the TFS rates at 6 (76.98%, 95% CI 65.79% to 86.65%) and 12 months (64.79%, 95% CI 50.20% to 78.19%).

**Conclusion:**

Patients with mNSCLC were able to achieve ongoing responses after discontinuation of ICI. In conclusion, the results of this meta-analysis indicate that different treatment regimens, different drugs or different treatment durations may have an impact on TFS.

## Background

Based on the recent cancer statistics from the American Cancer Society, lung cancer is a highly fatal disease that is still the leading cause of cancer mortality. Lung cancer remains the global public health priority due to high incidence, early malignancy and poor survival (1). Accounting for nearly 85% of lung cancer, non-small cell lung cancer (NSCLC) is the most common type of lung cancer, characterized by the most deadly malignancies and the lowest five-year survival rates (2). For patients with metastatic NSCLC (mNSCLC), cytotoxic chemotherapeutic drugs and microtubule stabilizing drugs are first line choice and have caused unprecedented prolonged survival (3–6). The discovery of driver genes and their tyrosine kinase inhibitors (TKI) has also led to new treatment strategies for mNSCLC patients (7, 8). Because of the lack of mutations in driver genes in wild-type mNSCLC, TKI does not improve patient outcomes well (9). In recent years, with the research on immune checkpoints and inhibitors such as programmed cell death protein 1/ligand 1 (PD-1/PD-L1) pathway or cytotoxic T lymphocyte-associated antigen 4 (CTLA-4), immunotherapy has shown good promise in the treatment of mNSCLC (10). Immune checkpoint inhibitor (ICI) stimulates the immune system to release potent T cells, so as to eliminate cancer cells (11). Although studies have shown that ICI does not exert significant efficacy in NSCLC with driver mutations (12, 13), however, new evidence suggests the emergence of ICI has dramatically altered the management and prognosis of wild-type mNSCLC and enable greater possibility of long-term survival (3–6, 14–17).

Due to the unique antitumor mechanism of ICI, patients treated with ICI achieve long-term therapeutic effects after discontinuation of ICI without the need for continued treatment or subsequent systemic therapy (11). In recent years, some studies have found that patients with mNSCLC can achieve long-term disease remission after discontinuing treatment with ICI (18, 19). Discontinuation of ICI therapy may be a potentially viable treatment option (20). Clinical studies have mainly used progression-free survival (PFS) and overall survival (OS) as endpoints for efficacy assessment, but this may not fully assess the outcome after discontinuation of ICI therapy (21). Recently, a new outcome measure, treatment-free survival (TFS), has been proposed, which may be more suitable for the estimation and comparison of clinical trials involving immuno-oncology drugs (22, 23). TFS is able to describe the durability of treatment benefit with no extension of maintenance therapy or subsequent initiation of systemic therapy (22). Currently, examining the ability to maintain response after discontinuation of ICI therapy, increasing the proportion of patients who achieve durable responses, and determining the optimal duration of therapy to balance efficacy, toxicity, and cost remain the focus of cancer immunotherapy. Studies of TFS rates may be extremely helpful in obtaining maximum therapeutic benefit and determining the optimal timing of treatment cessation. This finding has been studied and confirmed in metastatic melanoma and metastatic renal cell carcinoma (22–24), Although studies have been proposed for durable response after ICI discontinuation, there is still no relevant research to confirm and analyze the relevant influencing factors in NSCLC (20). This analysis aimed to assess the TFS after treatment discontinuation in patients with NSCLC treated with ICI who demonstrated partial or complete responses and to investigate and analyze factors that may mirror the objective response rate (ORR) and TFS.

## Materials and methods

This systematic review and meta-analysis was conducted in accordance with the Preferred Reporting Items for Systematic Reviews and Meta-Analyses statement (25).

### Search strategy and study selection

From the database inception to July 10, 2022, query from PubMed, Embase, and Cochrane library using the following algorithm: (“Non-Small-Cell Lung” OR “Non-Small Cell Lung” OR NSCLC) AND (immunotherapy OR “immune checkpoint” OR ipilimumab OR Atezolizumab OR Tislelizumab OR Pembrolizumab OR Nivolumab) AND (stop OR stopped OR discontinuation OR discontinue OR withdrawal OR withdrawn OR “treatment free”) AND (metastatic OR advanced). Additionally, we also manually searched the proceedings of important oncology conferences and study articles from other studies.

Prospective studies were included that met the following criteria: (1) patients diagnosed with mNSCLC and age ≥18 years of age; (2) patients in the study received ICI monotherapy or combination with other therapies; (3) reported the number of objective responders in at least a cohort of patients; (4) reported TFS in patients after discontinuation of ICI in at least one cohort of objective responders; (5) English literature; and (6) articles included at least one cohort with ≥12 months of follow-up. TFS was defined as the period from treatment discontinuation to subsequent systemic therapy initiation, death, or censoring, whichever occurred first (23, 24). Retrospective studies, case reports, systematic reviews, meta-analyses, letters, conference abstracts, or guidelines were excluded from the studies. If the results of the same study were described in multiple articles, only the most recently published and largest sample size was included in the statistics. Two reviews (Hu Y, Liu S) independently screened titles and abstracts according to the inclusion criteria, assessed full-text articles. All conflicting results were resolved by consensus.

### Data collection and quality assessment

Two authors (Hu Y, Liu S) independently extracted the following data from the articles that met the criteria: first author; publication or presentation year; clinical trial identifier and phase; immunotherapy regimen; discontinuation criteria (**Supplementary Table 1**); number of patients; patient characteristics (median age, percentage of male patients, performance status, programmed death ligand 1 (PD-LI) levels, smoking history, central nervous system (CNS)/brain metastasis, and systemic therapy history); duration of treatment; number of patients with sustained response to treatment; duration of follow-up and ORR. We also extracted all swimmer plots from the included study articles. WebPlotDigitizer V4.6 was used to extract TFS date from these plots for patients needed for analysis.

Newcastle-Ottawa scale was used to evaluated the risk of bias included articles. Based on the needs of this meta-analysis, Newcastle-Ottawa scale was modified by both authors (Hu Y, Liu S), meanwhile, each part of the included studies was evaluated as an independent cohort. The adjusted criteria were as follows: (1) cohort representing patients with advanced/metastatic NSCLC; (2) receipt of ICI monotherapy or in combination with other therapies; (3) demonstration that the outcome of interest was not present at the start of the study; (4) assessment of outcome using objectively defined criteria; (5) adequate follow-up time (≥12 months); and (6) adequate follow-up cohort (<10% lost to follow-up). Cohorts that met at least four criteria were considered high quality based on the quality assessment criteria used in the previous meta-analyses (26, 27).

### Statistical analysis

All data analyses in the study were performed using the meta (28) and metafor (29) package in R version R 4.2.0 and R Studio 2022.2.3. For all included cohorts, we calculated the proportion of patients with objective reflectivity, and we also calculated the proportion of patients with TFS at 6 and 12 months, along with the associated 95% CI. The Freeman-Tukey double-arcsine transformation (30) was chosen for the observed queue data for the calculation. We calculated pooled effect values using a random effects model (restricted maximum likelihood approach) and applied the inverse variance method to assign weights. Heterogeneity among cohorts was quantified by using I^2^ statistics. I^2^ > 75% indicated a high degree of heterogeneity (31). Two-sided P values<0.05 were considered statistically significant. A leave-one-out sensitivity analysis was used for all cohorts. Visual inspection of funnel plots, and Egger’s regression test were used to examine publication bias (32, 33).

## Results

### Search results

**Figure 1:**
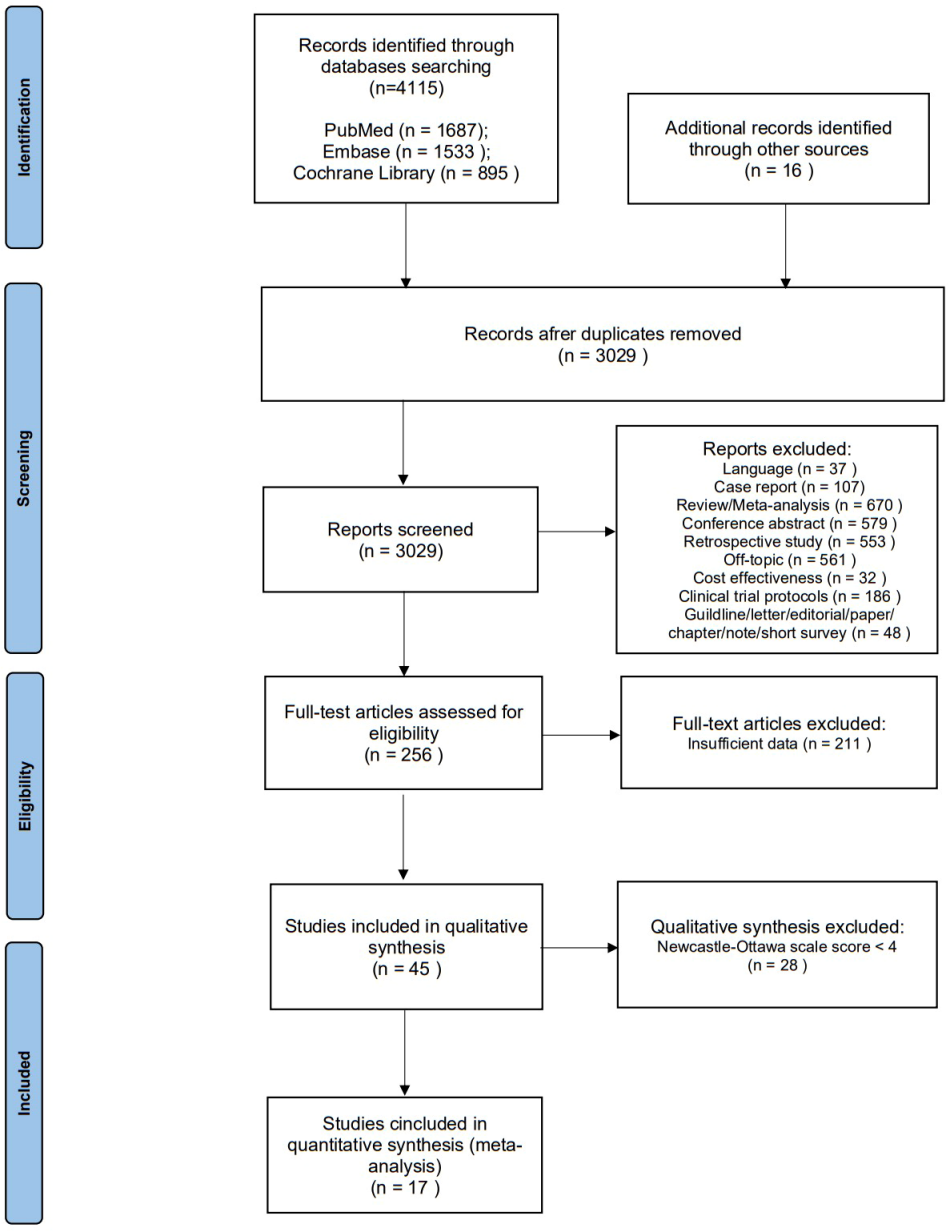
Preferred Reporting Items for Systematic Reviews and Meta- Analyses flow diagram for study selection.

We searched a total of 4115 articles from the systematic database, and 16 articles were identified by manual review of the reference lists. Of these, 1102 were excluded due to duplication, and after screening titles and abstracts, 2773 were excluded due to language, publication type, or topic. After full-text reading of the remaining 256 publications, 211 were excluded due to a lack of critical data. We assessed the quality of the resulting 45 publications, and 28 were excluded because of the Newcastle-Ottawa scale score <4. As a result, the final quantitative pooled analysis included 17 articles published between 2016 and 2022 (**Figure 1**) (13, 19, 34–48). Six of these articles included two separate cohorts (37, 39, 40, 42, 44, 47) and one article included four separate cohorts (35). Therefore, a total of 26 cohorts were analyzed in this meta-analysis (**Table 1**).

**Table 1:** Characteristics of included cohorts.

Author	Year	Trial identifier	Trial phase	Treatment	N	Median treatment duration (months)	Median follow-up (months)	Newcastle-Ottawa Scale score*
Awad et al (36)	2020	KEYNOTE021-G	2	Pembrolizumab+ Pem-Carb	60	–	49.4‡	4
Chen et al (42)	2020	NCT02582125	2	Nivolumab	11	3.3	–	5
Chen et al (42)	2020	NCT02582125	2	Nivolumab	41	3.3	–	5
Garon et al (39)	2019	KEYNOTE-001	1	Pembrolizumab	101	3.3&	60.6$	5
Garon et al (39)	2019	KEYNOTE-001	1	Pembrolizumab	449	3.3&	60.6$	5
Gettinger et al (13)	2016	CheckMate 012	1	Nivolumab	52	–	14.3	5
Herbst et al (19)	2020	KEYNOTE-010	2/3	Pembrolizumab	690	3.5	42.6	5
Horinouchi et al (37)	2019	ONO-4538‐05	2	Nivolumab	35	3.7	36§	6
Horinouchi et al (37)	2019	ONO-4538‐06	2	Nivolumab	76	2.5	36§	6
Horn et al (40)	2017	CheckMate 057	3	Nivolumab	292	2.6	24.2†	5
Horn et al (40)	2017	Checkmate 017	3	Nivolumab	135	3.2	24.2†	5
Lee et al (44)	2018	NCT02175017	2	Nivolumab	44	2.5	8.9	5
Lee et al (44)	2018	NCT02175017	2	Nivolumab	56	2.5	12.3	6
Masuda et al (45)	2022	UMIN000029602	2	Pembrolizumab	26	–	15.1	6
Naing et al (48)	2019	IVY	1b	Pembrolizumab/Nivolumab+Pegilodecakin	34	–	26.9	6
Nishio et al (38)	2018	KEYNOTE-025	1b	Pembrolizumab	38	–	19.2	6
Paz-Ares et al (47)	2022	CheckMate 227	3	Nivolumab+Ipilimumab	396	–	54.8#	4
Paz-Ares et al (47)	2022	CheckMate 227	3	Nivolumab+Ipilimumab	187	–	54.8#	4
Reck et al (46)	2021	CheckMate 9LA	3	Nivolumab+Ipilimumab+chemotherapy	361	6.1	30.7	5
Reck et al (41)	2021	KEYNOTE024	3	Pembrolizumab	154	7.9	59.9‡	4
Rizvi et al (35)	2016	CheckMate 012	1	Nivolumab+Gem-Cis	12	–	19¶	5
Rizvi et al (35)	2016	CheckMate 012	1	Nivolumab+Pem-Cis	15	–	19¶	5
Rizvi et al (35)	2016	CheckMate 012	1	Nivolumab+Pac-Carb	15	–	19¶	5
Rizvi et al (35)	2016	CheckMate 012	1	Nivolumab+Pac-Carb	14	–	19¶	5
Rodríguez-Abreu et al (34)	2021	KEYNOTE-189	3	Pembrolizumab+ Pem-Cis/Carb	410	7.2	31	4
Topalian et al (43)	2019	CA209-003	1	Nivolumab	129	3.1	58.3†	5

*Modified for a maximum score of 6, with studies scoring 4 or above considered higher quality.

Carb, carboplatin; Cis, cisplatin; Gem, gemcitabine; Pac, paclitaxel; Pem, pemetrexed.

†Minimum follow-up.

‡Median time from randomization to data cutoff.

&Median follow-up time for all patients in KEYNOTE-001.

$Median treatment duration time for all patients in KEYNOTE-001.

§Approximate follow-up time.

#Median follow-up time for all patients in CheckMate 227.

¶Median follow-up time for all patients in CheckMate 012(Naiyer A 2016).

### Study and patient characteristics

The characteristics of the included patient cohorts were shown in the **Table 1** and **2**. Overall, 3833 patients (26 cohorts) were included in the meta-analysis, 16 cohorts were ICI monotherapy (13, 19, 37–45), 4 cohorts were dual ICI (46–48), 6 cohorts were ICI plus chemotherapy (34–36). The median age of all patients ranged from 58 to 78 years. The proportion of male patients was higher, accounting for 65.1%. Also, 99.5% of patients had an Eastern Cooperative Oncology Group (ECOG) score of 0-1, approximately 14,6% had CNS/brain metastasis, and about 82% of patients had a history of smoking. According to the Newcastle-Ottawa scale, the included cohorts were highly methodological quality (**Table 1**).

**Table 2:** Baseline patient characteristics.

Author	Median age (years)	Male	ECOG 0-1	PD-L1 TPS				Smoking status current or former/never/unknown (%)	Prior systemic treatments (n)	CNS/Brain metastasis (n)
				<1%	1%-49%	≥50%	unknow/not quantifiable/Indeterminate			
Awad et al (36)	62.5 (40-77)	22 (37%)	59 (98%)	35%	32%	33%	0%	75/unknow/unknow	–	12 (20%) †
Chen et al (42)	60 (44-72)	10 (90.9%)	11 (100%)	45.5%	27.3%*	–	27.3%	90.9/9.1/0	≥0	1 (9.1%)
Chen et al (42)	62 (24-84)	22 (53.7%)	41 (100%)	61.5%	10.3*	–	28.2%	24.4/75.6/0	≥0	4 (9.8%)
Garon et al (39)	68 (39-93)	60 (59%)	101 (100%)	–	–	–	–	89/11/0	≥0	–
Garon et al (39)	62 (28-85)	229 (51%)	447 (99%)	–	–	–	–	72/28/0	≥1	–
Gettinger et al (13)	67 (43-85)	26 (50%)	52 (100%)	27%	38%	23%	12%	79/21/0	≥0	–
Herbst et al (19)	–	425 (61.6%)	686 (99.7%)	0%	58%	42%	0%	81.9/17.8/0.3	≥1	104 (15.1%)
Horinouchi et al (37)	65 (31-85)	32 (91.4%)	35 (100%)	11%	29%	14%	46%	97.2/2.9/0	1-2	3 (8.6%)
Horinouchi et al (37)	64 (39-78)	49 (64.5)	76 (100%)	17%	26%	9%	47%	72.4/27.6/0	1-2	21 (27.6%)
Horn et al (40)	61 (37-84)	151 (52%)	292 (100%)	37%	42%*	–	21%	79/20/1	≥1	34 (12%)
Horn et al (40)	62 (39-85)	111 (82%)	133 (99%)	40%	47%*	–	13%	90/7/3	≥1	9 (7%)
Lee et al (44)	69.5 (40-80)	44 (100%)	44 (100%)	–	–	–	–	97.7/2.3/0	≥1	10 (22.7%)
Lee et al (44)	63.5 (29-77)	34 (60.7%)	56 (100%)	–	–	–	–	62.5/37.5/0	≥1	16 (28.6%)
Masuda et al (45)	78 (75-90)	18 (69.3%)	26 (100%)	0%	0%	100%	0%	80.7/19.3/0	≥0	–
Naing et al (48)	67 (56-74)	18 (53%)	34 (100%)	38%	9%	21%	32%	–	≥0	2 (6%)
Nishio et al (38)	66 (41-78)	26 (68%)	38 (100%)	0%	68%	32%	0%	66/34/0	≥1	6 (16%) †
Paz-Ares et al (47)	64 (26-84)	255 (64.4%)	395 (99.7%)	0%	48.2%	51.8%	0%	84.3/14.1/1.5	≥0	41 (10.4%)
Paz-Ares et al (47)	63 (34-87)	260 (65.5%)	186 (95.5%)	100%	0%	0%	0%	87.2/12.3/0.5	≥0	23 (12.3%)
Reck et al (41)	64.5 (33-90)	92 (59.7%)	153 (99.4%)	0%	0%	100%	0%	96.8/3.2/0	≥0	18#
Reck et al (46)	65 (35-81)	252 (70%)	360 (99%)	37.4%	35.5%	21.1%	6.1%	87/13/0	–	65 (18%)
Rizvi et al (35)	67 (49-76)	7 (58%)	12 (100%)	–	–	–	–	100/0/0	≥0	–
Rizvi et al (35)	60 (34-78)	6 (40%)	15 (100%)	–	–	–	–	80/20/0	≥0	–
Rizvi et al (35)	58 (34-69)	7 (47%)	15 (100%)	–	–	–	–	80/13/7	≥0	–
Rizvi et al (35)	64 (47-83)	6 (43%)	14 (100%)	–	–	–	–	79/21/0	≥0	–
Rodríguez-Abreu et al (34)	65 (34-84)	254 (62%)	406 (99%)	31.0%	31.2%	32.2%	5.6%	88.3/11.7/0	≥0	73 (17.8%)
Topalian et al (43)	65 (38-85)	79 (61.2%)	127 (98.4%)	–	55.9%	19.1%	–	–	≥1	–

Data are presented as median (range) or number of patients (%), unless otherwise stated.

CNS, central nervous system; ECOG, Eastern Cooperative Oncology Group; PD-L1, programmed death ligand 1; TPS, tumor proportion score.

*Including ≥50% of patients.

#Treated brain metastases.

†Stable brain metastasis.

### ICI treatment and ORR

**Figure 2:**
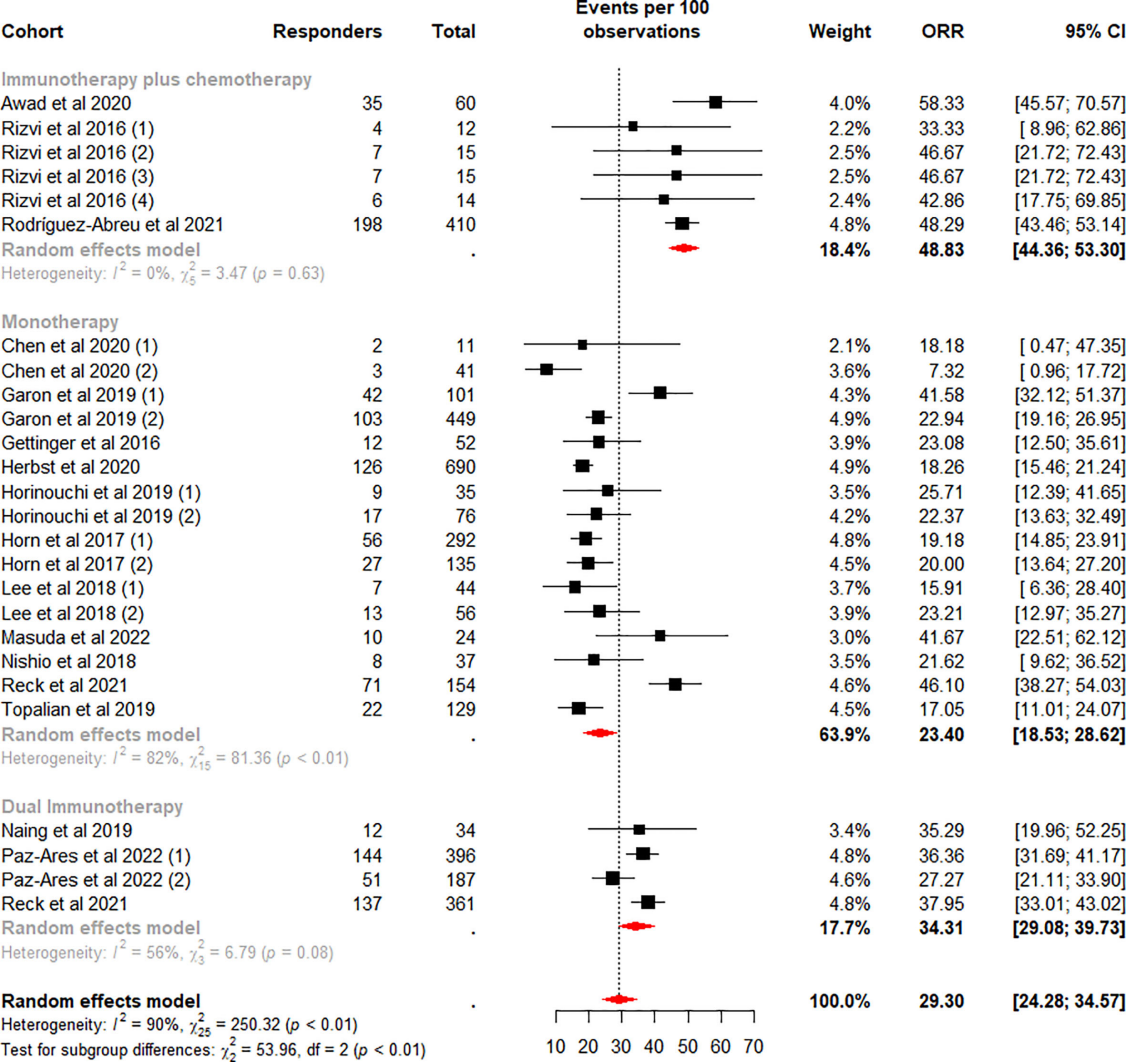
Random-effects (RE) meta-analysis of objective response rate (ORR) in patients with metastatic non-small cell lung cancer treated with immune checkpoint inhibitors (ICI) stratified by ICI regimen type. Total: number of response-evaluable patients; Events per 100 observations: confirmed ORR (%).

The weighted mean ORR for mNSCLC patients who were treated with ICI was 29.30% (95% CI 24.28% to 34.57%) (**Figure 2**), with significant heterogeneity between cohorts (I^2^ = 90%, p<0.01). Sensitivity analysis (**Supplementary Figure 1A**) by removing one cohort at a time showed that no outlier cohorts were identified and that the estimated total proportion was not affected by any of the cohorts.

**Figure 3:**
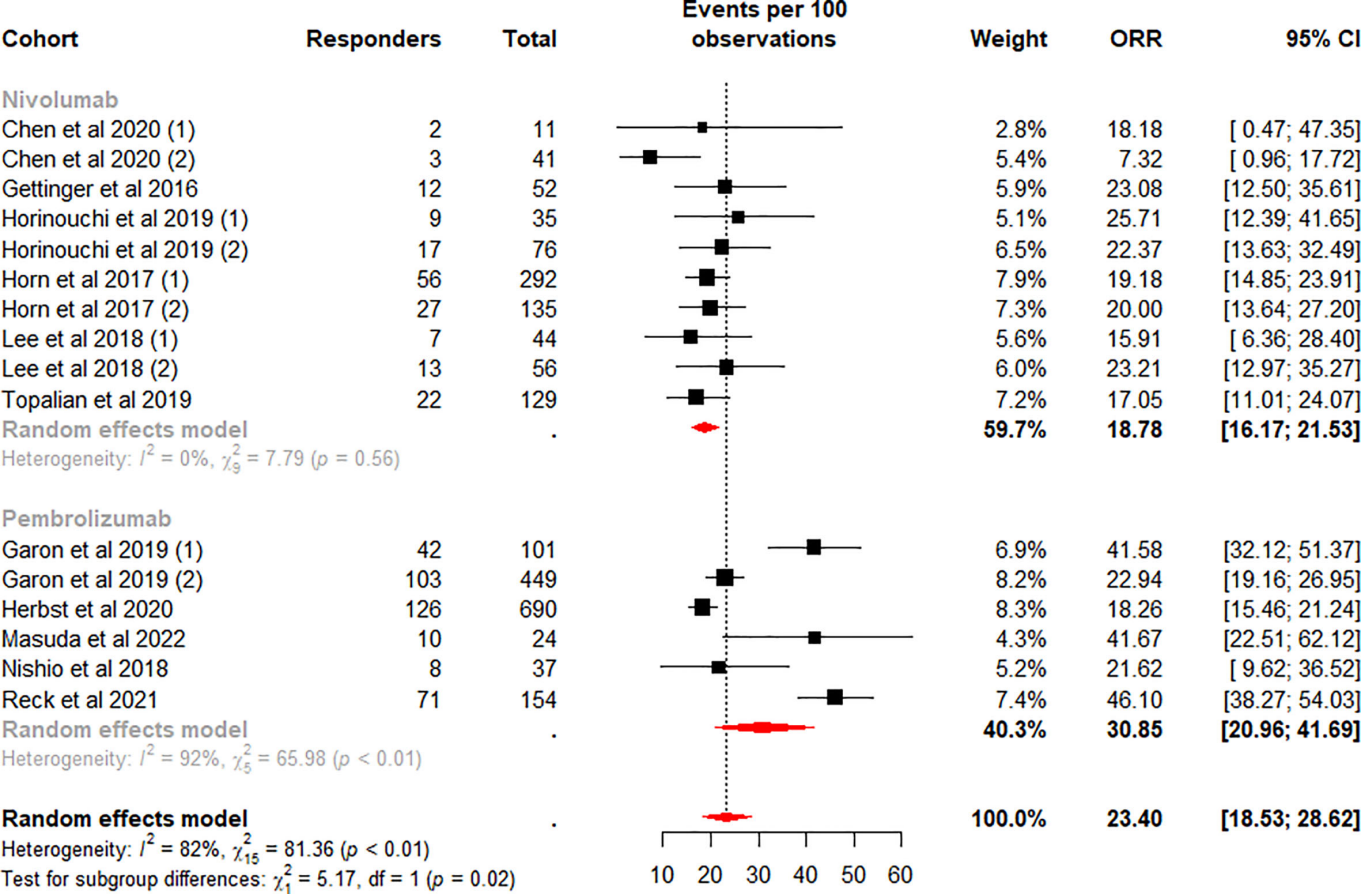
Random-effects (RE) meta-analysis of objective response rate (ORR) in patients with metastatic non-small cell lung cancer treated with immune checkpoint inhibitors (ICI) monotherapy stratified by drug type. Total: number of response-evaluable patients; Events per 100 observations: confirmed ORR (%).

Subgroup analysis was performed according to the ICI regimen type. Among the subgroups, the difference in the pooled ORR was highly significant (p<0.01). The highest weighted mean ORR was observed for ICI plus chemotherapy (48.83%, 95%CI 44.36% to 53.30%), and the lowest weighted mean ORR was observed for monotherapy (23.40%, 95%CI 18.53% to 28.62%). Meanwhile, heterogeneity was low and moderate for ICI plus chemotherapy (I^2^ = 0%, p = 0.63) and dual ICI (I^2^ = 56%, p = 0.08) and high for the monotherapy subgroup (I^2^ = 82%, p<0.01). We considered whether the higher heterogeneity of monotherapy was due to different drugs, so we performed a subgroup analysis on this again. Data analysis showed a lower weighted mean ORR and lower within-group heterogeneity for Nivolumab (18.78%, 95%CI 16.17% to 21.53%, I^2^ = 0%), in contrast, a higher weighted mean ORR and higher within-group heterogeneity for the Pembrolizumab subgroup (30.85%, 95%CI 20.96% to 41.69%, I^2^ = 92%) (**Figure 3**). Our analysis of the higher heterogeneity may be due to different intervention/treatment designs across cohorts. Although the source of heterogeneity in the Pembrolizumab subgroup was not found in the study, these results indicate that drug type still has some degree of influence on ORR.

### ICI treatment and TFS

A total of 26 cohorts were further analyzed to better characterize the outcome of discontinuing ICI in objective responders (13, 19, 34–48). Of these cohorts, 19 cohorts reported swimmer plots for all patients in complete or partial responders in the study (13, 35, 37–40, 42–45, 48), 4 cohorts reported swimmer plots for patients in complete or partial responders who completed 35 courses of treatment (19, 34, 36, 41), and 3 cohorts reported patients with swimmer plots from patients in complete or partial responders who discontinued treatment due to treatment-related adverse events (TRAEs) (46, 47).

We reviewed articles related to cohorts other than the 19 cohorts and found subsets of studies with two cohorts (49, 50) that reported swimmer plots including all patients in complete or partial responders in the subset, with similar levels of patients at baseline and all patients at baseline in both subset cohorts (**Supplementary Tables 2**, **3**). Ultimately, a number of 21 cohorts (395 patients) was included in the analysis (13, 35, 37–40, 42–45, 48–50). Of these patients, discontinuation of ICI therapy was documented in 194 cases, with a median TFS range of 0.0 to 25.4 months, and approximately 35.5% of patients demonstrated an ongoing response after discontinuation of ICI therapy (**Table 3**). Of note, one cohort was missing patients who discontinued ICI therapy (35), so this cohort was excluded from the analysis. A sensitivity analysis of cohorts with removing one cohort at a time showed that no outlier cohorts were found and the estimated sum cohorts were not influenced by individual cohorts (**Supplementary Figures 2A**, **3A**).

**Table 3:** Treatment-free survival after discontinuation of immune checkpoint inhibitors in all patients with objective response.

Study	Trial identifier	Responders(n)	Responders who discontinued ICI(n)	Median TFS (months)	Ongoing response off-treatment (%) *
Naing et al., 2019 (48)	IVY	12	9	9.4	33.3
Chen et al., 2020 (42)	NCT02582125	2	1	2.3	–
Chen et al., 2020 (42)	NCT02582125	3	2	0.3	–
Garon et al., 2019 (39)	KEYNOTE-001	42	20	9	–
Garon et al., 2019 (39)	KEYNOTE-001	103	50	12.5	–
Gettinger et al., 2016 (13)	CheckMate 012	12	7	4	71.4
Horinouchi et al., 2019 (37)	ONO-4538‐05	9	4	25.4	50
Horinouchi et al., 2019 (37)	ONO-4538‐06	17	8	6.9	37.5
Horinouchi et al., 2021 (49)	KEYNOTE-189(Japan Study)	14	10	1.3	20
Horn et al., 2017 (40)	CheckMate057	56	22	2.1	13.6
Horn et al., 2017 (40)	Checkmate017	27	6	1.9	33.3
Lee et al., 2018 (44)	NCT02175017	7	4	0.6	0
Lee et al., 2018 (44)	NCT02175017	13	4	1.7	25
Masuda et al., 2022 (45)	UMIN000029602	10	4†	10.7	75
Nishio et al., 2018 (38)	KEYNOTE-025	8	7§	9.5	85.7
Reck et al., 2016 (35)	CheckMate 012	4	0	0	0
Reck et al., 2016 (35)	CheckMate 012	7	1	8	0
Reck et al., 2016 (35)	CheckMate 012	7	1	20.4	100
Reck et al., 2016 (35)	CheckMate 012	6	3	2.3	66.7
Satouchi et al., 2021 (50)	KEYNOTE-024(Japan Study)	14	14	11.5	28.6
Topalian et al., 2019 (43)	CA209-003	22	17§	13.3	41.2

*Of responders who discontinued ICI.

§Does not include patients who discontinued ICI following progressive disease, as study did not report whether subsequent systemic therapy was started.

†Does not include patients who discontinued ICI following adverse event, as study did not report whether subsequent systemic therapy was started.

ICI, immune checkpoint inhibitors; TFS, treatment-free survival in responders who discontinued ICI.

**Figure 4:**
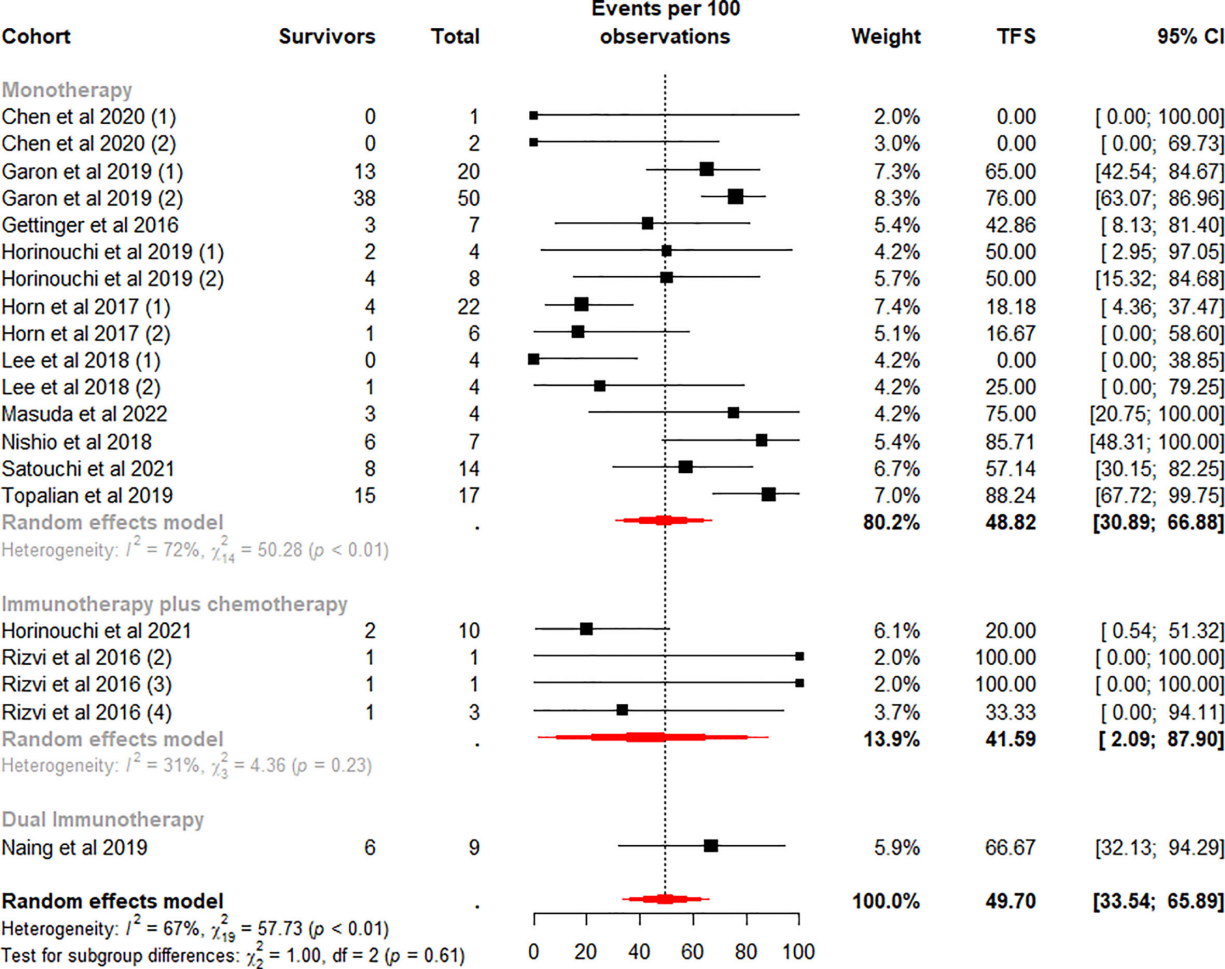
Random-effects (RE) meta-analysis of 6-month treatment-free survival (TFS) rate in patients with metastatic non-small cell lung cancer treated with immune checkpoint inhibitors (ICI) stratified by ICI regimen type. Total: number of responders who discontinued ICI; Events per 100 observations: TFS rate (%).

**Figure 5:**
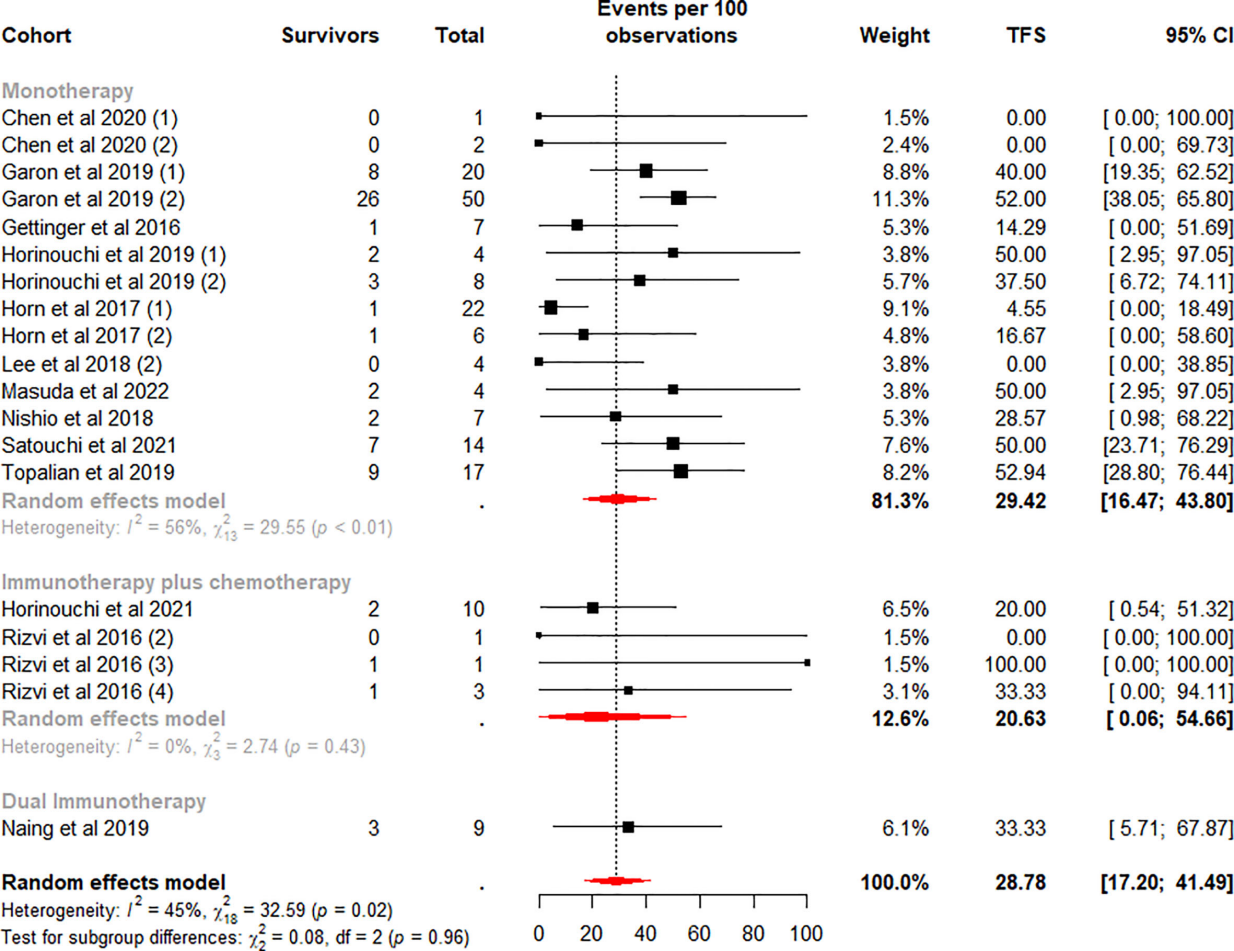
Random-effects (RE) meta-analysis of 12-month treatment-free survival (TFS) rate in patients with metastatic non-small cell lung cancer treated with immune checkpoint inhibitors (ICI) stratified by ICI regimen type. Total: number of responders who discontinued ICI; Events per 100 observations: TFS rate (%).

The TFS rates in the patients who discontinued ICI treatment significantly differed at 6 and 12 months, with a weighted mean TFS of 49.70% (95% CI 33.54% to 65.89%, I^2^ = 67%, p<0.01) and 28.78% (95% CI 17.20% to 41.49%, I^2^ = 45%, p=0.02), which showed some heterogeneity between cohorts (**Figures 4**, **5**).

To further analyze the effect of different factors on efficacy, we stratified the study cohort according to the type of ICI regimen and performed subgroup analyses. Analysis showed that pooled TFS rates based on ICI regimens were not found to be significantly different between the 6-month (p=0.61) and 12-month (p=0.96) subgroups (**Figures 4**, **5**). Besides, compared with the ICI plus chemotherapy subgroup, the monotherapy subgroup had moderate heterogeneity in TFS at either 6 (I^2^ = 72%, p<0.01) or 12 (I^2^ = 56%, p<0.01) months, suggesting a potential difference between cohorts in this subgroup.

**Figure 6:**
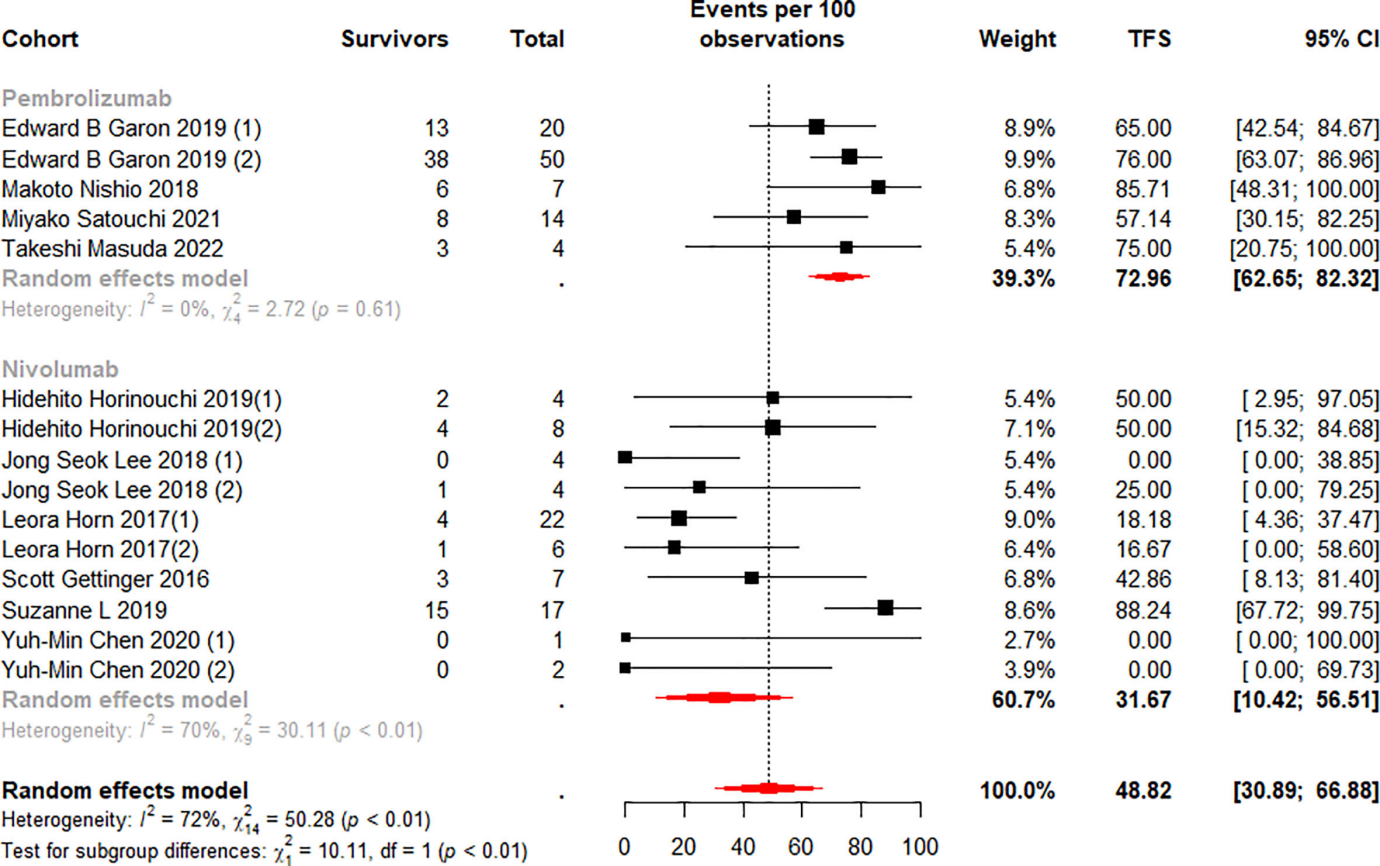
Random-effects (RE) meta-analysis of 6-month treatment-free survival (TFS) rate in patients with metastatic non-small cell lung cancer treated with immune checkpoint inhibitors (ICI) monotherapy stratified by drug type. Total: number of responders who discontinued ICI; Events per 100 observations: TFS rate (%).

**Figure 7:**
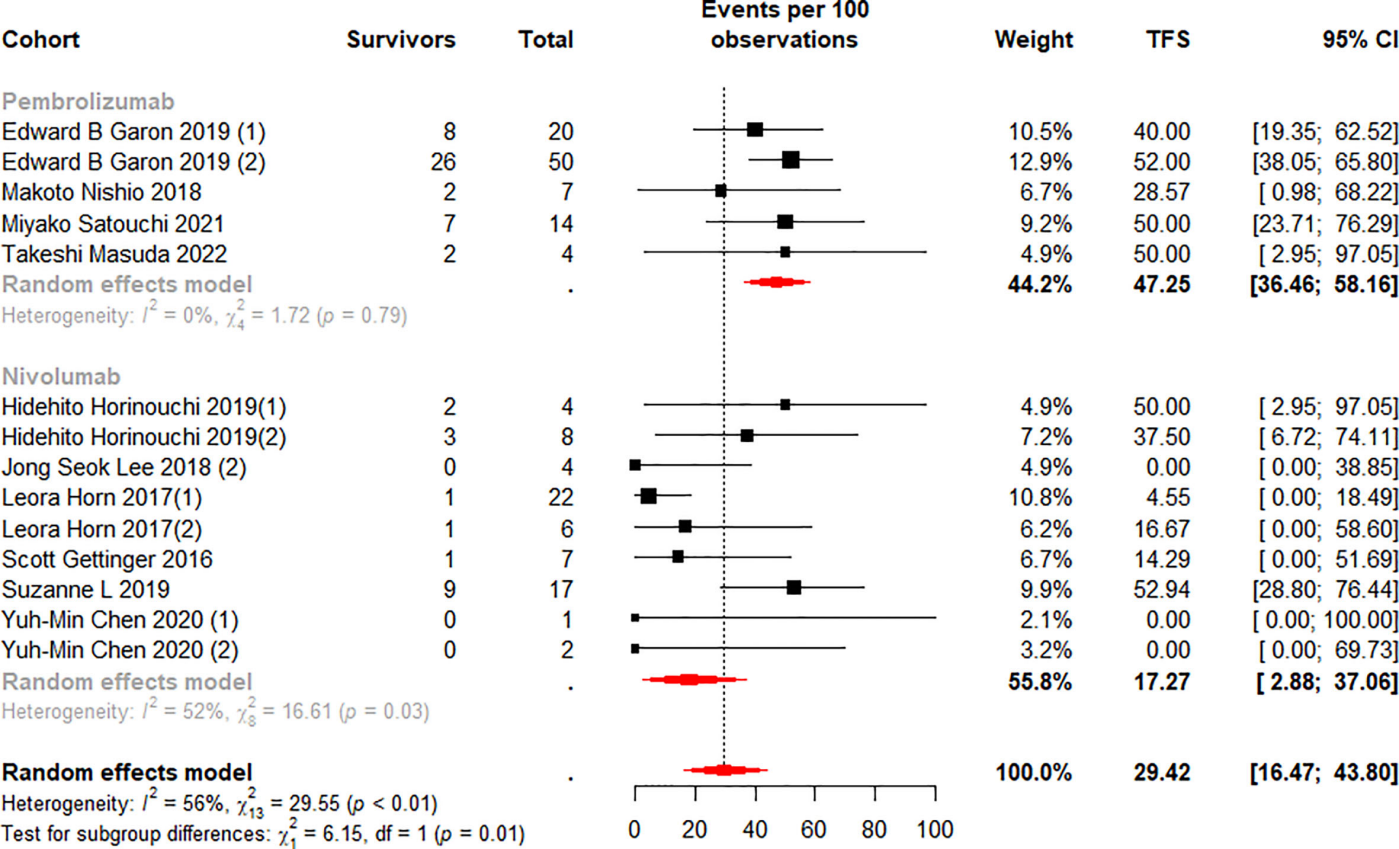
Random-effects (RE) meta-analysis of 12-month treatment-free survival (TFS) rate in patients with metastatic non-small cell lung cancer treated with immune checkpoint inhibitors (ICI) monotherapy stratified by drug type. Total: number of responders who discontinued ICI; Events per 100 observations: TFS rate (%).

We stratified the monotherapy subgroups again by drug class, and 15 cohorts (13, 37–40, 42–45, 50) were included in the analysis. Subgroup analysis showed significant differences between the Pembrolizumab and Nivolumab subgroups at 6 (p<0.01) and 12 (p=0.01) months (**Figures 6**, **7**). Notably, the weighted mean TFS rates were significantly higher in patients treated with Pembrolizumab (72.96%, 95% CI 62.65% to 82.32% for 6-month TFS rate; 47.25%, 95% CI 36.46 to 58.16% for 12-month TFS rate) than in the Nivolumab treatment subgroup (31.67%, 95% CI 10.42% to 56.51% for 6-month TFS rates; 17.27%, 95% CI 2.88% to 37.06% for 12-month TFS rates) at 6 and 12 months. (**Figures 6**, **7**). While TFS rates in patients treated with Pembrolizumab did not show significant within-subgroup variability (I^2^ = 0% at both 6 and 12 months), TFS rates in patients receiving Nivolumab showed moderate heterogeneity at 6 months (I^2^ = 70%, p<0.01) and 12 months (I^2^ = 52%, p=0.03).

### Complete 35 cycles of treatment

A total of 186 patients (4 cohorts) were included in the analysis, all of whom completed 35 cycles of treatment with Pembrolizumab alone or combination chemotherapy (19, 34, 36, 41). Patients included in the cohort had similar baseline levels to all patients (**Supplementary Tables 4**, **5**). No outlier cohort found in sensitivity analysis (**Supplementary Figure 1B**). Of these patients, 167 achieved complete or partial response with a weighted mean ORR of 91.15% (95%CI 82.50% to 97.33%) and a moderate level of heterogeneity between cohorts (I^2^ = 60%, P=0.06) (**Supplementary Figure 4**).

Discontinuation of ICI therapy was documented in 154 of 167 patients with a median TFS range of 5.3 to 25.7 months (**Supplementary Table 6**). One cohort was found to have an outlier TFS at 12 months at the time of examination (34), as well as this cohort was judged to be an outlier in the sensitivity analysis and was not included in the study when calculating the 12-month TFS rate (**Supplementary Figures 2B**, **3B**).

The pooled TFS rates were significantly different at 6 and 12 months with 80.18% (95% CI 53.03% to 97.87%, I^2^ = 89%, p<0.01) and 66.98% (95% CI 46.90% to 84.47%, I^2^ = 74%, p=0.02), respectively, with significant heterogeneity between cohorts (**Supplementary Figure 5**). Heterogeneity might have arisen from differences in treatment regimens across the cohorts. Overall, patients who completed 35 cycles of treatment had a considerably higher incidence of TFS at 6 and 12 months than all patients treated.

### Discontinue due to TRAEs

An additional analysis of patients who discontinued ICIs due to TRAEs was performed, which included a total of 3 cohorts (158 patients) (46, 47) with baseline patient characteristics generally consistent with the overall study population (**Supplementary Tables 7**, **8**). The weighted mean ORR (n=81) for these patients was 51.27% (95% CI 43.37% to 59.15%) (**Supplementary Figure 6**), with no significant differences found among the cohorts (I^2^ = 0%, p=0.91). Although patients discontinued ICI treatment due to the development of TRAEs, 32.1% of responders showed an ongoing response after discontinuation of ICI (**Supplementary Table 9**). At 6 and 12 months, the analysis found that their pooled TFS rates showed relatively high levels of 76.98% (95% CI 65.79% to 86.65%) and 64.79% (95% CI 50.20% to 78.19%) (**Supplementary Figure 7**), with low heterogeneity between cohorts (I^2^<50% for all TFS rates).

## Publication bias

All pooled analyses estimated publication bias by constructing funnel plots (**Supplementary Figure 8**) and Egger’s regression test. Funnel plot symmetry and Egger’s regression test P>0.05 were considered to have no significant publication bias. For ORR, funnel plot and Egger’s regression test (p=0.70) suggested no publication bias. Similarly, for patients in the whole treatment group, neither the funnel plot nor the Egger’s regression test for TFS rates at 6 (p=0.13) and 12 (p=0.29) months showed significant publication bias.

## Discussion

In recent years, immunotherapeutic agents targeting the immune checkpoint pathway have shown great promise in clinical trials and have been rapidly added to the clinical treatment of mNSCLC (51). Investigating the correlation between ICI treatment cessation time and efficacy remains the focal-point of current clinical trials.

We quantified the collected patient TFS data and identify four key findings. First, approximately 35.5% were able to obtain a long-term sustained response after ICI treatment (**Table 3**), and the mean incidence of TFS at 6 (49.7%) and 12 (28.78%) months was also high (**Figures 4**, **5**). Secondly, in the monotherapy subgroup analysis, patients treated with Pembrolizumab had higher ORR, as well as higher TFS at 6 and 12 months (30.85% vs 18.78% for ORR, 72.96% and 47.25% vs 31.67% and 17.27% for TFS), than those treated with Nivolumab (**Figures 3**, **6**, **7**). Third, for patients who completed 35 cycles of treatment with Pembrolizumab or combination with chemotherapy, the ORR (91.15%) and the 6-month and 12-month TFS rates exhibited high percentages, especially the 6-month (80.18%) and 12-month (66.98%) TFS rates were > 60% (**Supplementary Figures 4**, **5**). Finally, the data analysis revealed that 32.1% patients who discontinued ICI treatment due to TRAEs exhibited a durable response, and the 6 (76.98%) and 12 months (64.79%) TFS also remained at a high level, even closer to the TFS rates of patients who completed 35 cycles of treatment (**Supplementary Figure 7**).

Meanwhile, published studies about the TFS rate in ICI treatment for metastatic renal cell carcinoma showed that a clear inverse relationship existed between ORR and TFS in patients treated with ICI-based combinations (24). Interestingly, this present analysis found a positive relationship between ORR and TFS in patients treated with monotherapy. In the subgroup discussion of monotherapy, both ORR and TFS were significantly higher for patients treated with Pembrolizumab (30.85% for ORR, 72.96% and 47.25% for TFS) than for those treated with Nivolumab (18.78% for ORR, 31.67% and 17.27% for TFS). Notably, the pooled ORR of ICI combined with chemotherapy was higher than that of ICI alone (48.83% vs 23.40%), but no significant difference was found between ICI regimens in terms of TFS for both 6 months (p=0.61) and 12 months (p=0.96) TFS rates. Our current research results were consistent with previous findings which indicated that ICI plus chemotherapy had a better ORR in combination or not with other treatments in terms of mNSCLC immunotherapy (52, 53), but in monotherapy, Pembrolizumab patients performed better (54–57).

In addition, the findings presented in this paper can provide some confidence for clinical practitioners to discontinue Pembrolizumab or combination chemotherapy after 35 cycles. Some studies have proposed a second course of treatment after 35 cycles, such as the KEYNOTE-010 trial (Efficacy of Pembrolizumab versus docetaxel in mNSCLC) in which some recurrent patients underwent a second course of treatment (19), and analysis showed that 43% of patients achieved objective remission after treatment. While this confirms that recurrent patients can be re-treated with Pembrolizumab at progression and achieve disease control, the balance between efficacy and financial and toxicity remains a crucial consideration in the treatment of most chronic anticancer patients. In this analysis, the majority of patients who completed 35 cycles of Pembrolizumab or combination chemotherapy had PD-L1 TPS ≥1%, and since there are still no clear trials confirming the relationship between response rates and PD-L1 expression (58, 59), further studies are needed to determine whether similar therapeutic outcomes can be achieved for patients who do not express PD-L1 for this therapy.

In our analysis, we found that the 6 and 12 months TFS rates for patients who discontinued ICI therapy due to TRAEs were extremely similar to those of patients who completed 35 cycles of therapy, while 32.1% of patients also achieved a sustain response after discontinuation of therapy. The importance of safety and discontinuation of treatment due to TRAEs has also been highlighted in published studies (46), and our analysis provides some evidence for TRAEs as one of the criteria for clinical discontinuation of ICI therapy. However, it is worth noting that patients in this cohort were all predominantly treated with dual ICI therapy, and the correlation between higher TFS and dual ICI regimens cannot be denied. In the discussion of treatment regimen subgroups, the dual ICI therapy-based subgroup was included in only one cohort (n=9), and although the TFS obtained from the analysis was only moderate (66.67% and 33.33%), chance cannot be ruled out. More prospective clinical trials are needed to determine the influencing factors associated with obtaining a higher TFS.

This systematic review and meta-analysis bears some limitations. At the time of enumeration, trials of prospective ICI for patients with mNSCLC that who were absent of TFS data were excluded (60, 61). Some subgroups lacked sufficient trials. When estimating the incidence of TFS, the dual ICI subgroup had only one cohort, which was not statistically significant. Meanwhile, there were only 4 cohorts in the subset of patients who completed 35 cycles of treatment with either Pembrolizumab or combination cheotherapy (19, 34, 36, 41). In the analysis of patients who discontinued ICI therapy due to TRAEs, only three cohorts were included in the trial (46, 47). Since ICI plus chemotherapy consists of four treatment regimens and dual ICI treatment-based comprised of three treatment regimens, significant heterogeneity in the trial designs should be taken into consideration. As the TFS was extracted from the swimmer plot in the published literature, the accuracy of the extracted data was limited by the resolution of the images. When selecting patients for inclusion, patients who responded to ICI but were in stable condition were not included in the statistics, and some patient data were unavailable. Individual discontinuation criteria varied among patients in different trials. Because the distinct clinical outcomes exist in patients who discontinued the drug due to disease progression or excessive toxicity and those who discontinue for other reasons, caution is needed when interpreting these data.

To sum up, this study confirmed sustained response in patients after ICI discontinuation and provides sufficient evidence that TFS may emerge as an endpoint discussion in clinical trials involving NSCLC-related immuno-oncology agents. However, optimal timing of discontinuation of different treatment regimens without compromising the outcome needs further investigated with the aim to reduce the occurrence of excessive toxicity, and to select patients for ICI discontinuation correctly.

## Interpretation

To our knowledge, this is the first systematic review and meta-analysis to investigate and analyze the relevance of TFS in mNSCLC patients treated with ICI. Some of the mNSCLC patients treated with ICI could achieve durable TFS after treatment discontinuation. In addition, the findings of the meta-analysis suggest that different ICI treatment regimens, different drugs or different treatment discontinuation time may have an influence on TFS and also, to some extent, provide the possibility of TFS as a new clinical trial endpoint.

## Data availability statement

The original contributions presented in the study are included in the article/**Supplementary Material**. Further inquiries can be directed to the corresponding author.

## Author contributions

YH guarantees the integrity of the work. YH, SL, LW, YZ designed the study. YH, SL ran the systematic search, collected the data, and performed the analysis. LW, YL, DZ, YZ discussed the results and critically reviewed the manuscript. All authors contributed to writing the manuscript, and all authors approved the manuscript. All authors contributed to the article and approved the submitted version.
