# Supplementary material for: Treatment-free survival after discontinuation of immune checkpoint inhibitors in mNSCLC: a systematic review and meta-analysis
Source: Front Immunol. 2023 Jul 13;14:1202822. doi: 10.3389/fimmu.2023.1202822 (PMC10373084; doi:10.3389/fimmu.2023.1202822)
Supplement: Supplementary file 1 [file DataSheet_1.docx]

Supplementary Material

Treatment-free survival after discontinuation of immune checkpoint inhibitors in mNSCLC: a systematic review and meta-analysis

**Yue Hu, MD^1^, Shan Liu, PhD^1^, Lixing Wang, MD^1^, Yu Liu, MD^1^, Duohan Zhang, MD^1^, Yinlong Zhao, PhD^1^**

^1^Department of Nuclear Medicine, The Second Hospital of Jilin University, Changchun 130041, China

*** Correspondence:**Yinlong Zhao, PhD
Email: yinlong@jlu.edu.cn
Tel.: +86-0431-81136750
Fax: +86-0431-81136750

**Supplementary table 1. Immunotherapy discontinuation criteria**

| **Author** | **Year** | **Trial identifier** | **Immunotherapy discontinuation criteria** |
| --- | --- | --- | --- |
| Awad et al | 2020 | KEYNOTE-021G | PD, unacceptable toxicity, consent withdrawal, investigator decision, maximum cycle |
| Chen et al | 2020 | NCT02582125 | PD, unacceptable AEs |
| Chen et al | 2020 | NCT02582125 | PD, unacceptable AEs |
| Garon et al | 2019 | KEYNOTE-001 | PD, unacceptable toxicity, consent withdrawal, investigator decision |
| Garon et al | 2019 | KEYNOTE-001 | PD, unacceptable toxicity, consent withdrawal, investigator decision |
| Gettinger et al | 2016 | CheckMate 012 | PD, unacceptable toxicity, consent withdrawal, loss to follow-up |
| Herbst et al | 2020 | KEYNOTE-010 | PD, unacceptable toxicity, consent withdrawal, investigator decision, loss to follow-up, intercurrent illness, noncompliance with study treatment/procedures |
| Horinouchi et al | 2019 | ONO-4538‐05 | PD, unacceptable toxicity, consent withdrawal, death |
| Horinouchi et al | 2019 | ONO-4538‐06 | PD, unacceptable toxicity, consent withdrawal, death |
| Horn et al | 2017 | CheckMate 057 | PD, unacceptable toxicity, protocol-defined reasons |
| Horn et al | 2017 | Checkmate 017 | PD, unacceptable toxicity, protocol-defined reasons |
| Lee et al | 2018 | NCT02175017 | PD, unacceptable AEs, consent withdrawal |
| Lee et al | 2018 | NCT02175017 | PD, unacceptable AEs, consent withdrawal |
| Masuda et al | 2022 | UMIN000029602 | PD, unacceptable AEs, consent withdrawal, investigator decision |
| Naing et al | 2019 | IVY | PD, unacceptable toxicity, consent withdrawal, study end |
| Nishio et al | 2018 | KEYNOTE-025 | PD, unacceptable toxicity, consent withdrawal, investigator decision, end of 2-year treatment, intercurrent illness |
| Paz-Ares et al | 2022 | CheckMate 227 | PD, unacceptable toxicity, end of 2-year treatment |
| Paz-Ares et al | 2022 | CheckMate 227 | PD, unacceptable toxicity, end of 2-year treatment |
| Reck et al | 2021 | CheckMate 9LA | PD, unacceptable toxicity, end of 2-year treatment |
| Reck et al | 2021 | KEYNOTE024 | PD, unacceptable AEs, consent withdrawal |
| Rizvi et al | 2016 | CheckMate 012 | PD, unacceptable toxicity, consent withdrawal |
| Rizvi et al | 2016 | CheckMate 012 | PD, unacceptable toxicity, consent withdrawal |
| Rizvi et al | 2016 | CheckMate 012 | PD, unacceptable toxicity, consent withdrawal |
| Rizvi et al | 2016 | CheckMate 012 | PD, unacceptable toxicity, consent withdrawal |
| Rodríguez-Abreu et al | 2021 | KEYNOTE-189 | PD, unacceptable AEs, consent withdrawal, investigator decision, intercurrent illness |
| Topalian et al | 2019 | CA209-003 | PD, CR, unacceptable toxicity, consent withdrawal |

AEs, adverse events; CR, complete response; PD, progressive disease

**Supplementary table 2. Characteristics of included subset cohorts**

| Author | Year | Trial identifier | Trial phase | Treatment | N | Median treatment duration (months) | Median follow-up (months) | Newcastle-Ottawa Scale score* |
| --- | --- | --- | --- | --- | --- | --- | --- | --- |
| Horinouchi et al^49^ | 2021 | KEYNOTE-189 Japan subset | 3 | Pembrolizumab+ Pem-Cis/Carb | 25 | 9 | 18.5‡ | 4 |
| Satouchi et al^50^ | 2021 | KEYNOTE-024 Japan subset | 3 | Pembrolizumab | 21 | 13.1# | 43.3‡ | 4 |

*Modified for a maximum score of 6, with studies scoring 4 or above considered higher quality.

Carb, carboplatin; Cis, cisplatin; Pem, pemetrexed.

‡ Median time from randomization to data cut-off

# Median treatment exposure as of the data cut‐off date

**Supplementary table 3. Baseline patient characteristics of included subset cohorts**

| Author | Median age(years) | Male | ECOG 0-1 | PD-L1 TPS | | | | Smoking status current or former/never/unknown (%) | Prior systemic treatments(n) | CNS/Brain metastasis(n) |
| --- | --- | --- | --- | --- | --- | --- | --- | --- | --- | --- |
|  |  |  |  | <1% | 1%-49% | ≥50% | unknow/not quantifiable /Indeterminate |  |  |  |
| Horinouchi et al^49^ | 64(34-77) | 19(76%) | 25(100%) | 56% | 24% | 16% | 4% | 72/28/0 | ≥0 | 4(16%) |
| Satouchi et al^50^ | 66(40-80) | 16(76%) | 21(100%) | 0% | 0% | 100% | 0% | 95/5/0 | ≥0 | 1(5%) |

Data are presented as median (range) or number of patients (%), unless otherwise stated.

CNS, central nervous system; ECOG, Eastern Cooperative Oncology Group; PD-L1, programmed death ligand 1; TPS, tumor proportion score.

**Supplementary table 4. Characteristics of included cohorts that the patients who completed 35 cycles of treatment**

| Author | Year | Trial identifier | Trial phase | Treatment | N | Median treatment duration (months) | Median follow-up (months) | Newcastle-Ottawa Scale score* |
| --- | --- | --- | --- | --- | --- | --- | --- | --- |
| Awad et al^36^ | 2020 | KEYNOTE021-G | 2 | Pembrolizumab+ Pem-Carb | 12 | - | - | 4 |
| Herbst et al^19^ | 2020 | KEYNOTE-010 | 2/3 | Pembrolizumab | 79 | - | 43.4 | 5 |
| Reck et al^41^ | 2021 | KEYNOTE024 | 3 | Pembrolizumab | 39 | - | 34.7† | 4 |
| Rodríguez-Abreu et al^34^ | 2021 | KEYNOTE189 | 3 | Pembrolizumab+ Pem-Cis/Carb | 56 | 25.6 | 31.5‡ | 4 |

*Modified for a maximum score of 6, with studies scoring 4 or above considered higher quality.

Carb, carboplatin; Cis, cisplatin; Gem, gemcitabine; Pac, paclitaxel; Pem, pemetrexed.

† Median (range) time from completion of 35 cycles to data cutoff

‡ Median time from randomization to data cutoff

**Supplementary table 5. Baseline patient characteristics of completed 35 cycles of treatment**

| Author | Median age(years) | Male | ECOG 0-1 | PD-L1 TPS | | | | Smoking status current or former/never/unknown (%) | Prior systemic treatments(n) | CNS/Brain metastasis(n) |
| --- | --- | --- | --- | --- | --- | --- | --- | --- | --- | --- |
|  |  |  |  | <1% | 1%-49% | ≥50% | unknow/not quantifiable /Indeterminate |  |  |  |
| Awad et al^36^ | 60(40-76) | 2(17%) | 12(100%) | 25% | 75%* | - | 0% | 75/unknown/unknown | - | 1(8%) † |
| Herbst et al^19^ | - | 53(67.1%) | 79(100%) | 0% | 26.6% | 73.% | 0% | 91.1/8.9/0 | ≥0 | 12(15.2%) |
| Reck et al^41^ | 61(43-80) | 25(64.1%) | 39(100%) | 0% | 0% | 100% | 0% | 94.9/5.1/0 | - | 9(23.1%) # |
| Rodríguez-Abreu et al^34^ | 65.5(42-82) | 33(58.9%) | 56(100%) | 10.7% | 28.6% | 55.4% | 5.4% | 91.1/8.9/0 | ≥0 | 6(10.7%) |

Data are presented as median (range) or number of patients (%), unless otherwise stated.

CNS, central nervous system; ECOG, Eastern Cooperative Oncology Group; PD-L1, programmed death ligand 1; TPS, tumor proportion score.

*Including ≥50% of patients

#Treated brain metastases

†Stable brain metastasis

**Supplementary table 6. Treatment-free survival after discontinuation of immune checkpoint inhibitors in patients who have completed 35 cycles of treatment with objective response**

| Study | Trial identifier | Responders(n) | Responders who discontinued ICI(n) | Median TFS  (months) | Ongoing response  off-treatment (%) * |
| --- | --- | --- | --- | --- | --- |
| Awad et al^36^ | KEYNOTE-021-G | 12 | 11 | 22.2 | 63.6 |
| Herbst et al^19^ | KEYNOTE-010 | 75 | 71 | 12 | - |
| Reck et al^41^ | KEYNOTE-024 | 32 | 32 | 25.7 | - |
| Rodríguez-Abreu et al^34^ | KEYNOTE-189 | 48 | 40 | 5.3 | - |

*Of responders who discontinued ICI.

ICI, immune checkpoint inhibitors; TFS, treatment-free survival in responders who discontinued ICI.

**Supplementary table 7. Characteristics of included cohorts that the patients who discontinued due to TRAEs**

| Author | Year | Trial identifier | Trial phase | Treatment | N | Median treatment duration (months) | Median follow-up (months) | Newcastle-Ottawa Scale score* |
| --- | --- | --- | --- | --- | --- | --- | --- | --- |
| Paz-Ares et al^47^ | 2022 | CheckMate 227 | 3 | Nivolumab+Ipilimumab | 66 | 3.2 | 49.4§ | 4 |
| Paz-Ares et al^47^ | 2022 | CheckMate 227 | 3 | Nivolumab+Ipilimumab | 31 | - | 49.4§ | 4 |
| Reck et al^46^ | 2021 | CheckMate 9LA | 3 | Nivolumab+Ipilimumab+chemotherapy | 61 | 4.4 | - | 4 |

TRAEs, treatment-related adverse events

*Modified for a maximum score of 6, with studies scoring 4 or above considered higher quality.

§Minimum follow-up for all randomized patients in CheckMate 227.

**Supplementary table 8. Baseline patient characteristics of discontinued due to TRAEs**

| Author | Median age(years) | Male | ECOG 0-1 | PD-L1 TPS | | | | Smoking status current or former/never/unknown (%) | Prior systemic treatments(n) | CNS/Brain metastasis(n) |
| --- | --- | --- | --- | --- | --- | --- | --- | --- | --- | --- |
|  |  |  |  | <1% | 1%-49% | ≥50% | unknow/not quantifiable /Indeterminate |  |  |  |
| Paz-Ares et al^47^ | 64(45-80) | 45(68.2%) | 66(100%) | 0% | 43.9% | 56.1% | 0% | 3/93.9/3 | - | 2(3%) |
| Paz-Ares et al^47^ | - | 23(74.1%) | 31(100%) | 100% | 0% | 0% | 0% | 3/97/0 | - | 1(3%) |
| Reck et al^46^ | 66(44-78) | 39(64%) | 61(100%) | 42.6% | 41% | 14.8% | 1.7% | 95/5/0 | - | 8(13%) |

Data are presented as median (range) or number of patients (%), unless otherwise stated.

CNS, central nervous system; ECOG, Eastern Cooperative Oncology Group; PD-L1, programmed death ligand 1; TPS, tumor proportion score, TRAEs, treatment-related adverse events.

**Supplementary table 9. Treatment-free survival after discontinuation of immune checkpoint inhibitors in patients who discontinued due to TRAEs**

| Study | Trial identifier | Responders(n) | Responders who discontinued ICI(n) | Median TFS  (months) | Ongoing response  off-treatment (%) * |
| --- | --- | --- | --- | --- | --- |
| Paz-Ares et al^47^ | CheckMate 227 | 35 | 35 | 31.4 | 37.1 |
| Paz-Ares et al^47^ | CheckMate 227 | 15 | 15 | 29.5 | 20 |
| Reck et al^46^ | CheckMate 9LA | 31 | 31 | 12.2 | 32.3 |

*Of responders who discontinued ICI.

ICI, immune checkpoint inhibitors; TFS, treatment-free survival in responders who discontinued ICI; TRAEs, treatment-related adverse events

**(A)
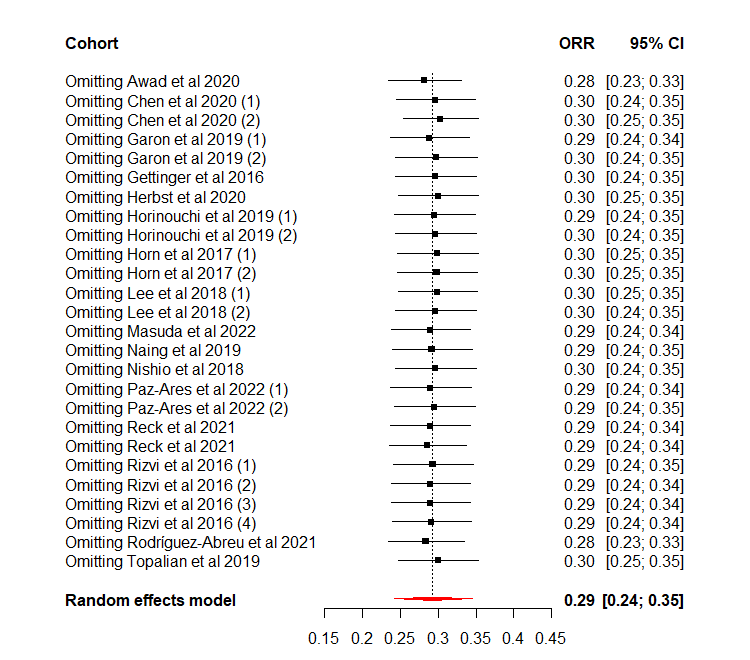
**

**(B)
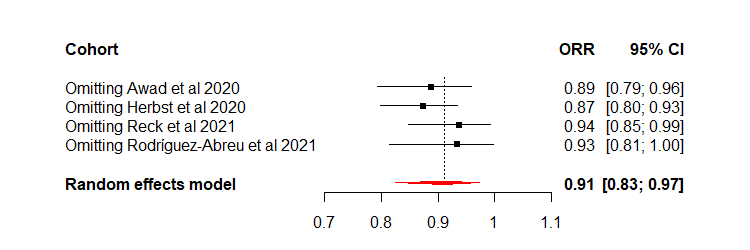
**

**(C)
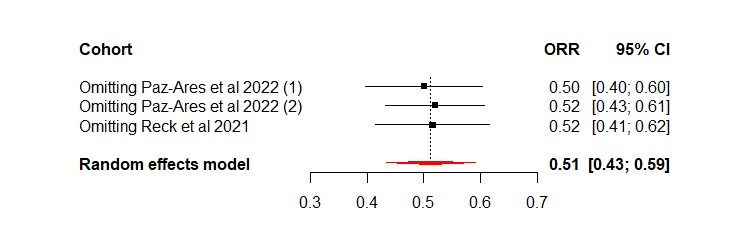
**

**Supplementary figure 1 - Forest plots depicting the results of leave-one-out sensitivity analysis, in which the summary proportion of ORR in ((A) all patients; (B) patients** **who have completed 35 cycles (~two years) of treatment; (C) patients who discontinued due to TRAEs) was estimated after iterative removal of each indicated study cohort**.

**(A)
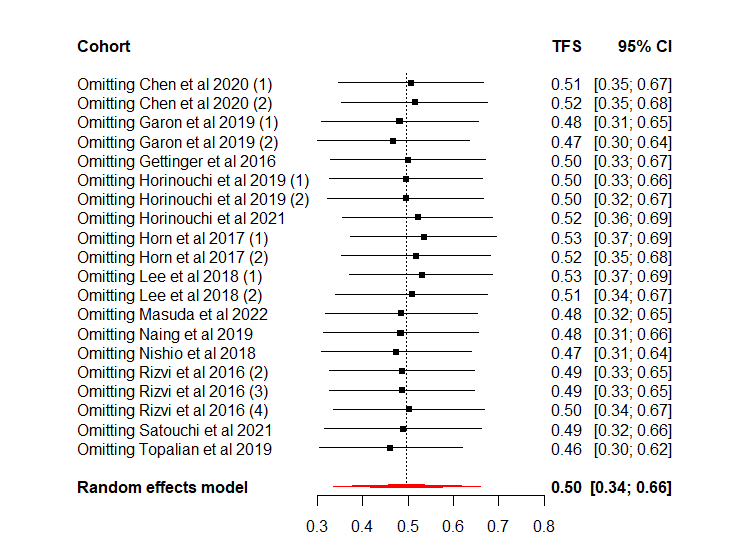
**

**(B)
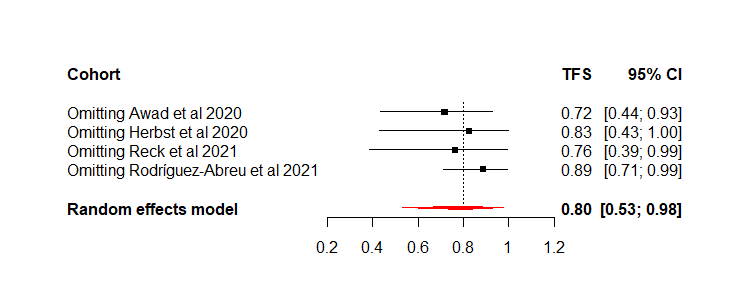
**

**(C)
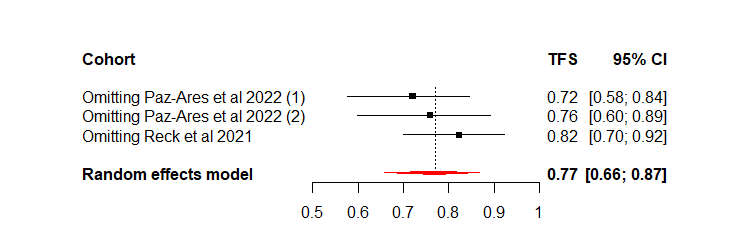
**

**Supplementary figure 2 - Forest plots depicting the results of leave-one-out sensitivity analysis, in which the summary proportion of TFS at 6 months in ((A) all patients; (B) patients who have completed 35 cycles (~two years) of treatment; (C) patients who discontinued due to TRAEs) was estimated after iterative removal of each indicated study cohort**.

**(A)
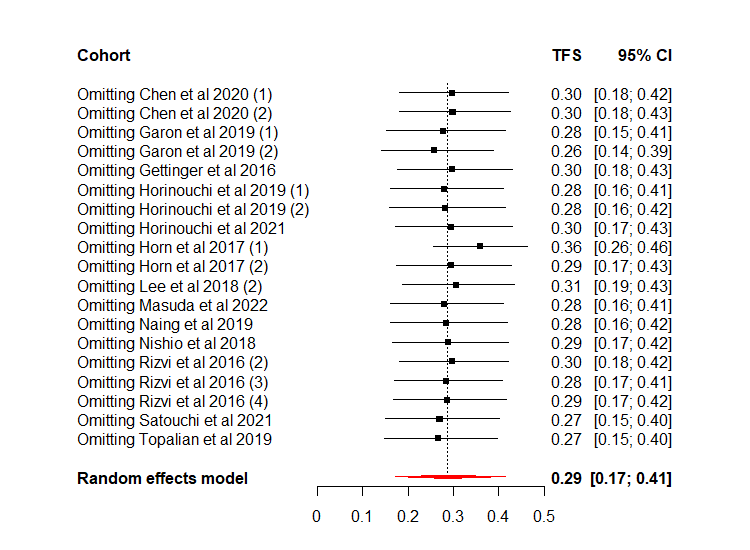
**

**(B)
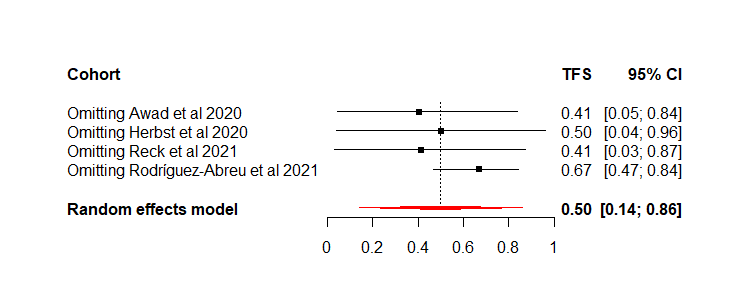
**

**(C)
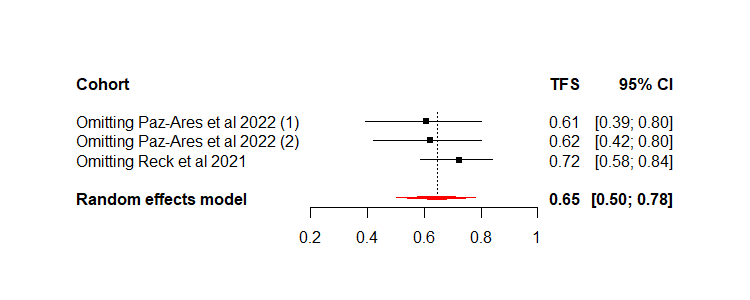
**

**Supplementary figure 3 - Forest plots depicting the results of leave-one-out sensitivity analysis, in which the summary proportion of TFS at 12 months in ((A) all patients; (B) patients who have completed 35 cycles (~two years) of treatment; (C) patients who discontinued due to TRAEs) was estimated after iterative removal of each indicated study cohort**.

**
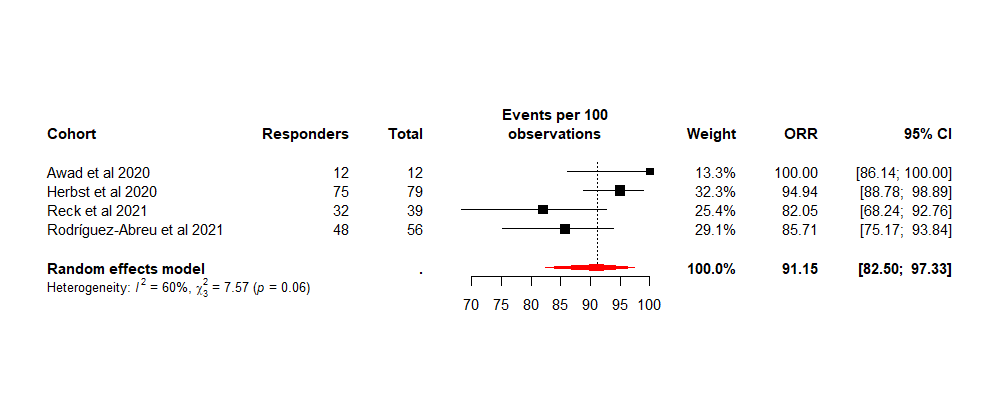
**

**Supplementary figure 4 - Random-effects (RE) meta-analysis of objective response rate (ORR) in patients who completed 35 cycles (~2 years) with metastatic non-small cell lung cancer treated with immune checkpoint inhibitors (ICI).**

Total: number of response-evaluable patients; Events per 100 observations: confirmed ORR (%).

**(A)
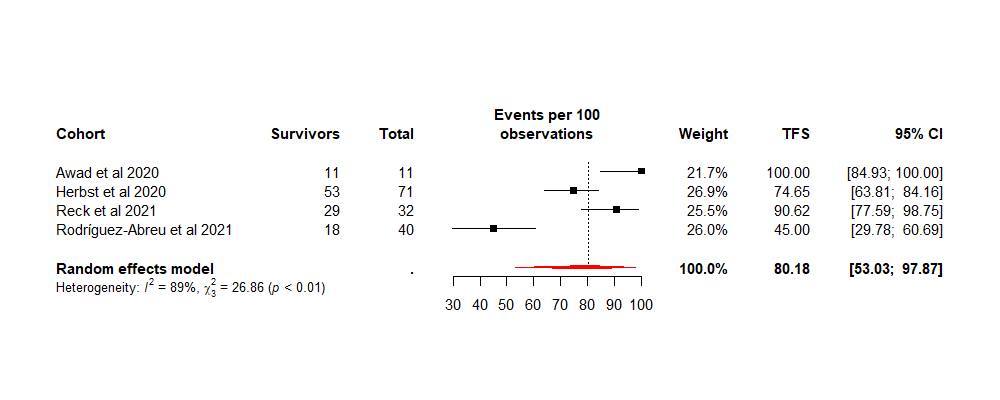
**

**(B)
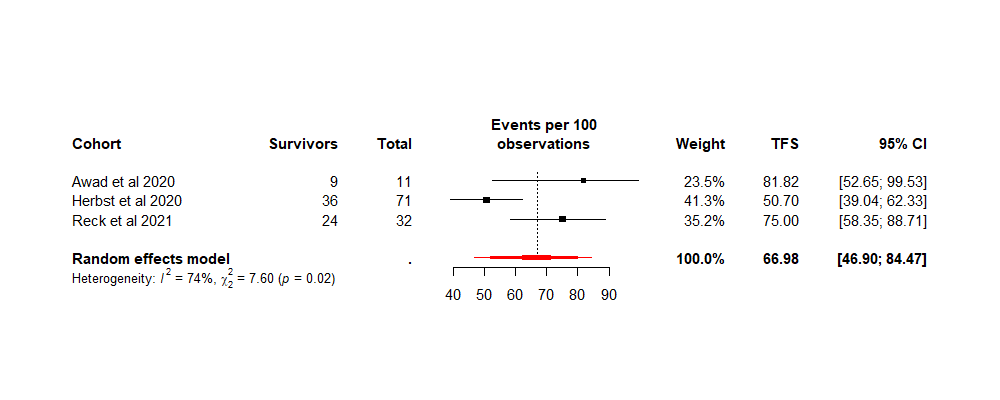
**

**Supplementary figure 5 - Random-effects (RE) meta-analysis of ((A) 6-month and (B)12-month) treatment-free survival (TFS) rate in patients who completed 35 cycles (~2 years) with metastatic non-small cell lung cancer treated with immune checkpoint inhibitors (ICI)**

Total: number of responders who discontinued ICI; Events per 100 observations: TFS rate (%).

**
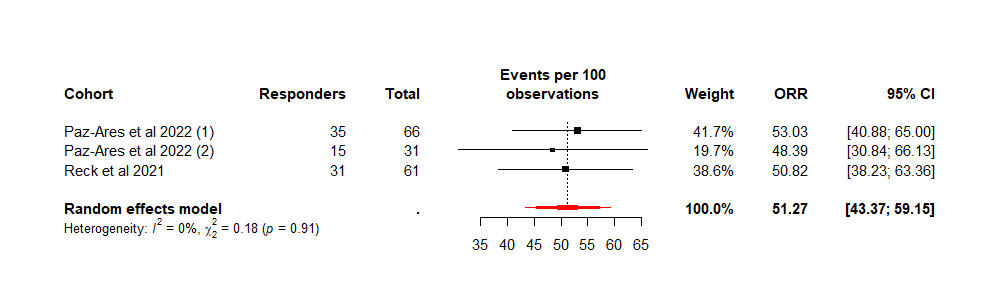
**

**Supplementary figure 6 - Random-effects (RE) meta-analysis of objective response rate (ORR) in patients who discontinued due to TRAEs with metastatic non-small cell lung cancer treated with immune checkpoint inhibitors (ICI).**

Total: number of response-evaluable patients; Events per 100 observations: confirmed ORR (%).

**(A)
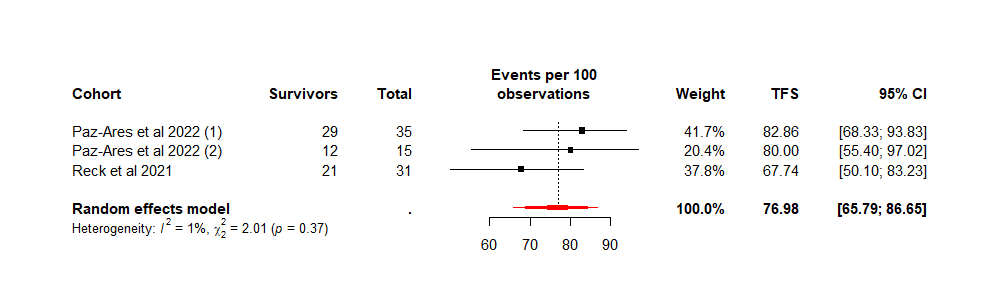
**

**(B)
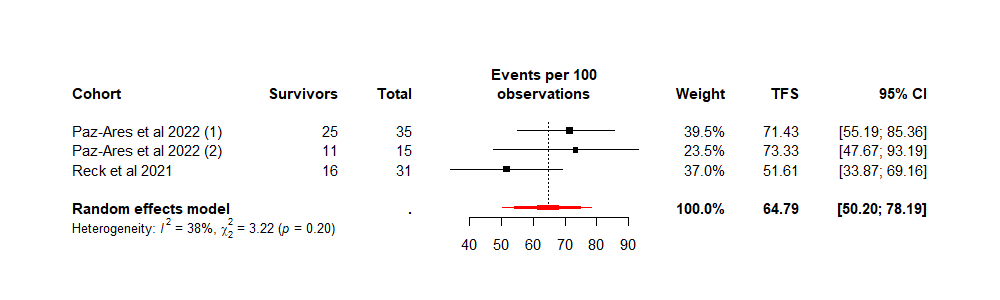
**

**Supplementary figure 7 - Random-effects (RE) meta-analysis of ((A) 6-month and (B)12-month) treatment-free survival (TFS) rate in patients who discontinued due to TRAEs with metastatic non-small cell lung cancer treated with immune checkpoint inhibitors (ICI).**

Total: number of responders who discontinued ICI; Events per 100 observations: TFS rate (%).

**(A)**

**
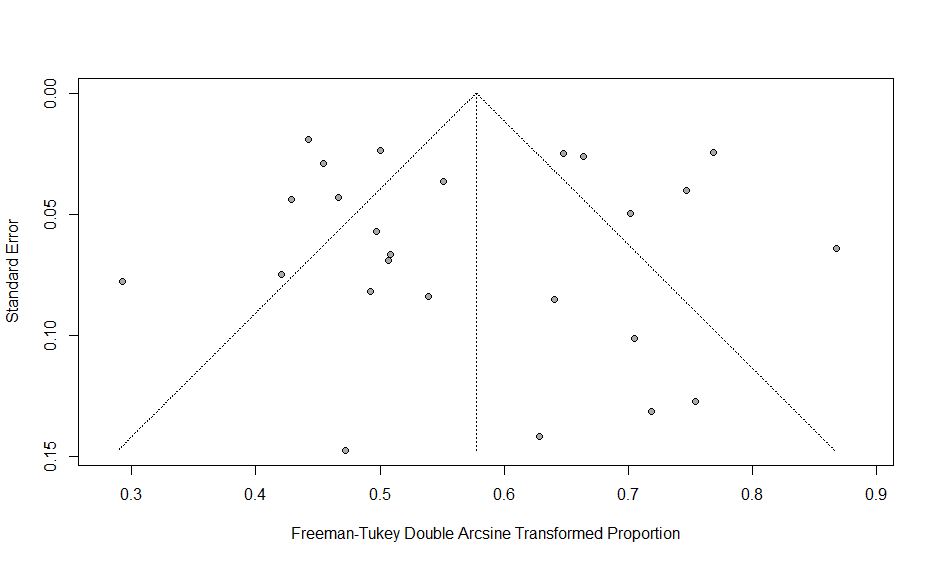
**

**(B)**

**
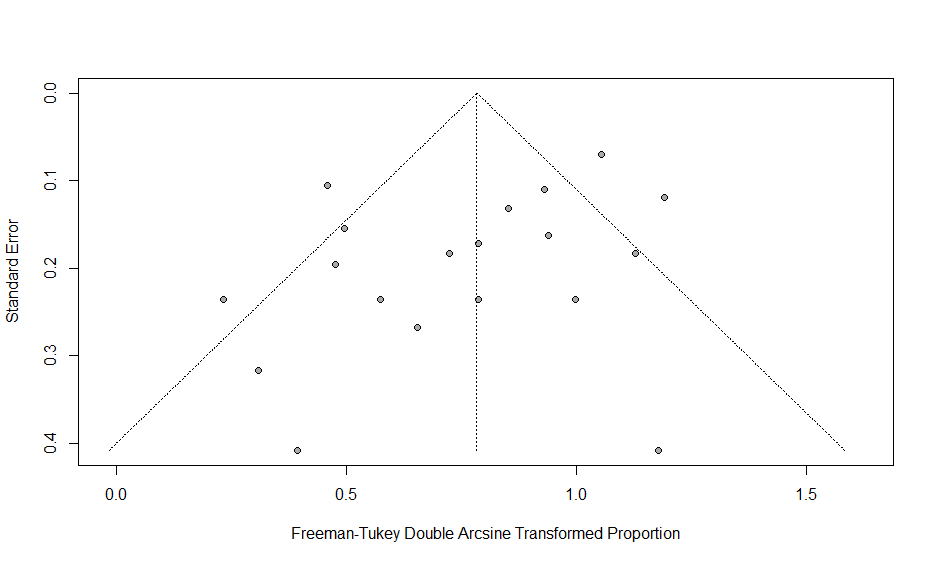
**

**(C)**

**
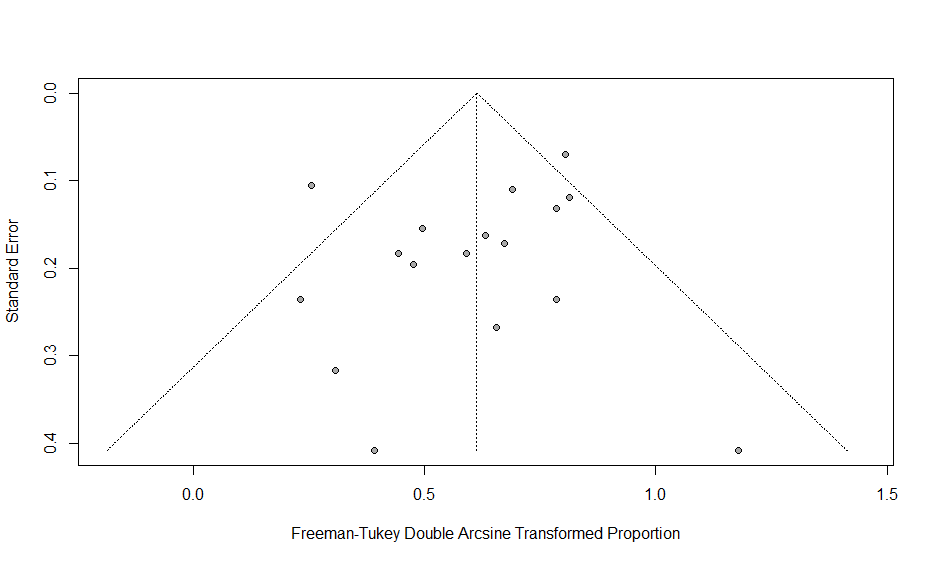
**

**Supplementary figure 8 - Funnel plots with pseudo 95% confidence limits in all patients for (A) ORR; (B) TFS rate at 6 months; (C) TFS rate at 12 months.**
